# Specific Resistance of Barley to Powdery Mildew, Its Use and Beyond: A Concise Critical Review

**DOI:** 10.3390/genes11090971

**Published:** 2020-08-21

**Authors:** Antonín Dreiseitl

**Affiliations:** Department of Integrated Plant Protection, Agrotest Fyto Ltd., Havlíčkova 2787, CZ-767 01 Kroměříž, Czech Republic; dreiseitl@vukrom.cz; Tel.: +420-573-317-139

**Keywords:** barley, *Blumeria graminis* f. sp. *hordei*, durability of resistance, *Hordeum vulgare*, powdery mildew, specific resistance

## Abstract

Powdery mildew caused by the airborne ascomycete fungus *Blumeria graminis* f. sp. *hordei* (*Bgh*) is one of most common diseases of barley (*Hordeum vulgare*). This, as with many other plant pathogens, can be efficiently controlled by inexpensive and environmentally-friendly genetic resistance. General requirements for resistance to the pathogens are effectiveness and durability. Resistance of barley to *Bgh* has been studied intensively, and this review describes recent research and summarizes the specific resistance genes found in barley varieties since the last conspectus. *Bgh* is extraordinarily adaptable, and some commonly recommended strategies for using genetic resistance, including pyramiding of specific genes, may not be effective because they can only contribute to a limited extent to obtain sufficient resistance durability of widely-grown cultivars. In spring barley, breeding the nonspecific *mlo* gene is a valuable source of durable resistance. Pyramiding of nonspecific quantitative resistance genes or using introgressions derived from bulbous barley (*Hordeum bulbosum*) are promising ways for breeding future winter barley cultivars. The utilization of a wide spectrum of nonhost resistances can also be adopted once practical methods have been developed.

## 1. Introduction

Barley (*H.v.* L.) is an important cereal crop and powdery mildew is one of its most frequent diseases [1,2] caused by the airborne ascomycete fungus *Blumeria graminis* (DC.) E. O. Speer, f. sp. *hordei* (*Bgh*) emend. É. J. Marchal (anamorph *Oidium monilioides* Link). This, as well as many other plant pathogens, can be efficiently controlled by inexpensive and environmentally-friendly genetic resistance [3]. However, *Bgh* is extraordinarily adaptable [4], and some commonly recommended strategies of using genetic resistance—for example, the development of specific resistance gene pyramids in host genotypes [5] or use of cultivars carrying specific resistance genes in varietal mixtures [6]—may be ineffective.

## 2. Specific Resistance

Qualitative specific genes condition the resistance of the host to avirulent pathotypes or susceptibility to virulent pathotypes of the pathogen. Based on the gene-for-gene model [7,8], specific genes were first postulated with pathogen isolates [9,10] and later identified with molecular markers [11,12] and sequencing [13]. To determine the effectiveness against the pathogen and for postulating specific genes, a large set of isolates with a broad spectrum of virulences/avirulences and their combinations is needed [14], whereas for studying the genetics of resistance, only a few isolates are necessary [15,16].

Identifying specific resistance genes by postulation is based on recording the responses of a variety after inoculation with pathogen isolates to obtain resistance type arrays (RTA). Comparing the RTA of a tested variety with RTAs of standard genotypes possessing known resistance genes can result in the postulation of known as well as unknown genes and their combinations [17,18]. It is not unusual to find five or more resistance genes present in one variety [19], and postulation is the most efficient method to analyze such complex data compared with other techniques such as genetic analyses, use of molecular markers or sequencing. New isolates introduced into resistance tests can aid the detection of other genes and supplement total numbers of identified genes and gene combinations.

### 2.1. Newly-Found Genes

A catalogue of specific powdery mildew resistance genes in European barley varieties has been reported [20], and known resistance genes in barley have been summarized in a conspectus [21] (Table 1). Other genes considered here as “recently described” were subsequently found mostly in cultivars and landraces (*H*. *vulgare* subsp. *vulgare*) and in wild barley (*H. vulgare* subsp. *spontaneum = Hvs*) (Table 2).

#### 2.1.1. Genes in Cultivars

Three recently described specific genes (*Ml(Ro)*, *MlaLv* and *Ml(Ve)*) have been widely used in commercial cultivars [34,35,36], and when these cultivars were marketed, the resistances were effective to almost all pathotypes [40]. However, directional selection soon resulted in the rapid reproduction of virulent pathotypes [41,42], and the cultivars became susceptible. *Ml(Ro)* originates from an *Hvs* accession 1B-53 [43], and *MlaLv* and *Ml(Ve)* were probably also derived from wild barley.

Another five recently described specific resistance genes have a negligible effect against the pathogen population. A knowledge of these resistances can help to deduce RTAs of tested varieties and provide data to confirm the pedigrees of cultivars. *Ml(Ch)* originally designated *Ml(SG)* [24] has a typical response type 2 (RT2) [44,45] (Supplementary Table S1) and is detected only after inoculation with an old Japanese isolate “Race I” [46]. This isolate is also avirulent to *Mla8* (RT0) [19,47] and, together with two known avirulent Israeli isolates, establishes an RTA typical of another recently described gene *MlaLo* [26,27]. Like *Mla8* and *Ml(Ch)*, *Ml(VIR)* has been also identified using only one known avirulent isolate [27]. *Ml(Dt6)* was confirmed as a sixth gene in Duet (besides *Ml* genes *a6*, *a14*, *g*, *CP* and *h*) several years after the registration of this cultivar. This was revealed when virulence frequencies to known resistance genes in a population did not explain the high avirulence frequency to resistance of this cultivar [24]. *Ml(Lu)* present in some winter barleys [19] was also detected several years after the postulation of other specific genes in these cultivars [10]. Another gene often present in cultivars carrying *Ml(Lu)* (e.g., in the German cultivar Borwina) is *Ml(Ru2)* [48,49] designated also as *Ml(Bw)* [43,50] and present in some European and frequently in southeast Asian barleys [51].

#### 2.1.2. Resistances Found in Landraces

The following five recently described genes were found in landraces, and despite the existence of some virulent isolates, all these genes were considered as potential resistance sources. *MlaN81* [22] used in the Czech cultivar Maridol [10] was not listed in the conspectus [21]. A gene was found in an accession SBCC097 of the Spanish Barley Core Collection and localized on chromosome 7HL [28]; it is designated here as *MlSb*. Two other genes (*MlMor* and *MlLa-H*) were found on chromosome 2HS [29] and 2HL [30], respectively, and the recessive gene *mlmr* was located on 6HL [31]. Furthermore, *Ml(Ln)* was detected in some European cultivars [25]; its origin is unknown but probably originates also from a landrace.

Many other sets of barley landraces collected in Tunisia [52], Morocco [53,54,55,56,57,58,59,60,61], Australia [62], China [51], Greece [63], Jordan [64], Latvia [65], Libya [66], Spain [28,67,68], Turkey [69,70] or from more than one area [71,72,73,74,75,76,77] have been studied, and numerous known and unknown specific resistances were characterized including accessions resistant to a wide range of pathogen isolates.

#### 2.1.3. The Great Diversity of Specific Resistances in Wild Barley

The first study of barley resistance against powdery mildew was performed on progenies from crossing *H*. *vulgare* with wild barley [78], which was later recognized as a large pool of resistance to powdery mildew [79,80,81]. The widespread use of specific resistances from *Hvs* in barley breeding started after genetically analyzing many accessions [82,83], and 13 genes had already been included in the conspectus [21]. Apart from these, four other genes originating from *Hvs* have been described, namely one designated as allele*32* at the *Mla* locus [32] and three genes (*Mlf*, *Mlj* and recessive *mlt*) at different loci [33], which could be combined with other genes including alleles of *Mla*. However, there were high virulence frequencies of a pathogen population collected from *Hvs* grown in Israel to most of these 17 genes [84].

In the following studies, many unidentified genes were described. In 20 accessions of *Hvs*, 39 specific genes were detected [85]. Many genes, which were different from those previously identified, were found among 24 lines derived from crosses between two winter barley cultivars and *Hvs* accessions [86], and a total of 27 genes were found in 15 of these lines [87]. Resistance among 116 accessions of *Hvs* from Israel and Jordan was detected in 58% and 70% of them, respectively [88].

Among 1383 accessions from the United States Department of Agriculture (National Small Grains Collection), 123 accessions were resistant to all 22 isolates that were mostly European [89], but only one of them was resistant after testing with 38 Israeli isolates [90]. Thirteen of these resistant accessions contained unidentified genes; one gene was found in five accessions, two genes in seven accessions and three genes in one accession [91]. Moreover, in seven accessions [12,16,92,93,94], dominant genes were located on chromosome 1HS (probably in *Mla* locus), and three accessions [12,93,94] contained dominant genes on chromosome 2HS. In three other accessions [12,92,95], genes on 7HS were detected, and in one accession [12], a gene on chromosome 7HL was localized.

More recently, 582 accessions from a *Hvs* collection of the ICARDA (International Centre for Agricultural Research in the Dry Areas) collected mostly in the Near East, were screened for resistance to powdery mildew [96,97,98]. In a set of 146 heterogeneous accessions represented by 687 plant progenies, only 56 progenies were susceptible to all 32 isolates used, 46 plants were resistant and 611 progenies exhibited RTAs indicating the presence of specific resistances and their combinations [98]. In all these studies of *Hvs*, it was reported that there was a huge diversity of specific resistances against powdery mildew.

### 2.2. Effectiveness of Specific Resistances

General requirements for resistance to plant pathogens are effectiveness and durability (effectiveness in time) [99,100,101]. Monogenic specific resistances to *Bgh*, particularly those newly-introduced, are often characterized by initial high effectiveness to almost the whole pathogen population in a specific area when virulence frequency is close to zero and often exhibit low Phenotypes (RTs) such as 0, 0–1 or 1 that do not allow even the limited reproduction of avirulent pathotypes [45]. Phenotypes (RTs) of resistance genes after inoculation of a variety with avirulent pathotypes are stable in these conditions. However, a population of *Bgh* is usually large, and mutations from avirulence to virulence frequently arise in these populations, or virulent pathotypes often migrate from other areas. As a consequence, cultivars with corresponding specific resistance genes induce directional selection and rapid reproduction of virulent pathotypes results in a loss of effectiveness and the collapse of resistance in the field. Hence, increasing virulence complexity against resistance genes in the cultivars and concurrent recombinations lead to greater population diversity and evolution of new pathotypes with a high potential for overcoming a wide range of resistances and their combinations [42].

*Mla8* is an example of a very effective resistance gene against avirulent pathotypes because it is typified by having the lowest phenotype (RT0—no traces of the pathogen with occasional slight necrosis after inoculation). However, *Mla8* could be detected only with a few old Japanese isolates [46] because in European and other world populations no avirulent pathotypes have been found. Therefore, in laboratory or greenhouse conditions, *Mla8* is highly effective against avirulent pathotypes, but in the field is ineffective and fully susceptible to “natural” populations of the pathogen.

Specific resistances that are initially effective soon become ineffective. One example is *MlaLv* designated according to winter barley cultivar Laverda [35], which was registered in Germany in 2005 and in the Czech Republic in 2007. The source of this gene is unknown, but it could be derived from a wild barley accession. No virulence to *MlaLv* was found among 160 isolates collected from the air across the Czech Republic in 2008 [102]. However, once Laverda and other varieties with this resistance began to be widely grown, virulent pathotypes emerged through mutation and migration, and the virulence frequency rapidly increased by directional selection. In the four years after detecting the first virulent isolate, this virulence was already present in more than half of the pathotypes [40], and cultivars with *MlaLv* were fully susceptible in the field.

This is another classical example loss of effectiveness, in this case on a well-documented continental scale. *Mla13* (RT0) was a very effective resistance against the European population of powdery mildew. First, cultivar Koral carrying *Mla13* was released in the Czech Republic in 1978, and soon, the gene was extensively used in European spring barleys [20]. By the end of 1980, the first virulent pathotype was found [103], and in 1985, a strong epidemic of powdery mildew occurred in the Czech Republic [2] mainly on spring cultivars with *Mla13*. Within three years, a strong infection of similar cultivars across Europe and the United Kingdom was recorded [104]. There are many examples of specific resistances being overcome in a similar way to *MlaLv* and *Mla13*, but no cases of widely-used specific genes maintaining sufficiently durable resistance have been reported.

The effectiveness of specific genes is related to corresponding virulence frequencies. There are several methods to study populations of plant pathogens [105], but using isolates derived from spores sampled from the atmosphere [42,106] is the most suitable for characterizing airborne pathogens. Nevertheless, there are some anomalies in the interpretation of results. If the virulence frequency to a resistance gene is low, then it is customary to state that the corresponding gene is effective. However, low virulence frequency can also indicate that the area under cultivation of cultivars carrying the resistance gene is also low. If this area is, for example, 1%, then a virulence frequency of 1% reflects an average infection of such cultivars compared with other cultivars grown in that location [36,107].

The limited effectiveness of specific resistances over a period (durability) [2,4,108] can be extended for the lifetime of individual cultivars by combining (pyramiding) several genes effective against the whole spectrum of pathotypes into one genotype [5,109]. Those resistances should not be used individually in other cultivars, since they will often be quickly overcome and render the gene combinations ineffective. Such a restriction is hard to implement in practice. Furthermore, there will be a great demand for new resistance sources including independently inherited genes for breeding only a few cultivars. To combine such resistances requires the availability and adoption of molecular markers tightly linked to the resistance loci. These requirements for pyramiding nondurable resistance genes against a pathogen are expensive, and obtaining a return on the investment is questionable, since durability of combined specific resistances even for the lifetime of individual cultivars is still not guaranteed.

### 2.3. Using Specific Resistances in Breeding Programmes and Research

The primary importance of resistance genes is protecting a host against pathogens. Complete effectiveness of specific resistances to avirulent pathotypes is commonly achieved as outlined above, but is soon overcome by virulent pathotypes. Despite this, a knowledge of specific resistance genes in cultivars has wider utilization in research and practical agriculture. Considering varietal resistance, selected cultivars can be used as differentials for studying pathogen populations to monitor changes including virulence frequencies to individual genes and their contribution to pathogen evolution [2]. Almost all current barley cultivars and many landraces and wild barley accessions contain one or more major and often specific resistance genes. Therefore, when looking for partial resistance [110,111,112], the response of specific genes that mask minor resistance genes must be well-characterized and challenged with corresponding virulent isolates prior to investigating the minor genes. Furthermore, knowledge of specific resistances is a valuable tool to establish authenticity and purity of cultivars and to confirm their pedigrees [77]. Results of testing landraces and wild barley can also be invaluable for mapping and determining the global distribution of native resistances. Nevertheless, based on existing experience, the use of specific resistance in breeding cannot be recommended because there is no way of exploiting it to achieve durable cultivar resistance.

## 3. The Future beyond Specificity

Specific genes have little importance for providing durable resistance of barley cultivars against powdery mildew. Nevertheless, our understanding of problems associated with other ways of breeding for durable resistance is increasing, and genetic resources and technical tools are becoming available.

### 3.1. MLO

MLO is based on a nonspecific recessive gene *mlo* that is one of only a few plant resistance genes effective against an entire pathogen species. Most high-yielding European spring barley cultivars carry this resistance [14,44]. In spite of this, it has been widely used for more than four decades [113] and is still a source of durable resistance for future spring barley cultivars. However, *mlo* should not be used for breeding winter barley because the pathogen does produce a few colonies of asexual spores on cultivars carrying this gene. Therefore, the presence of *mlo* in both spring and winter barley could result in the year-round adaptation and subsequent development of partial virulence and gradual erosion of the effectiveness of this unique resistance gene [113,114].

### 3.2. Quantitative Resistance

One option for breeding barley, especially winter cultivars, against powdery mildew attack is accumulating (pyramiding, stacking) nonhypersensitive, nonspecific quantitatively inherited resistance genes [110,115,116,117,118] originating from cultivars as well as landraces [119] and wild barley [97,98,120,121,122,123,124]. Although not all quantitative genes are necessarily nonspecific, this way shows promise. A similar approach has proved effective in intensively cultivated winter wheat maintained vegetatively for a prolonged period conducive for powdery mildew infection in the United Kingdom [108].

### 3.3. Resistant Introgressions from Bulbous Barley

Another solution mainly for winter barley could use resistances derived from bulbous barley (*Hordeum bulbosum* (*Hb*)) [125,126], which is the only representative in the secondary gene-pool of cultivated barley [127]. A series of *Hb* introgression lines (ILs) harbouring a diverse set of desirable resistance traits has been developed and is being routinely used as source of novel diversity in gene mapping studies [13,125,126]. Many of these ILs are freely available from the Nordic Genebank [128]. Some were tested with sets of mildew isolates, and one of them (181P94/1/3/1/1/1-2) was resistant to all isolates that were used [129,130]. The resistance is associated with an introgression on chromosome 2HS, and further molecular analyses and allelism tests with other 2HS ILs will determine whether different loci are involved [131].

Three resistance genes (*Mlhb1*, *Mlhb2* and *Mlhb.A42*) against the powdery mildew pathogen have been designated [13,37,38,39,132] (Table 2). However, the lack of recombination between the introgressed *Hb* fragments and orthologous chromosomes of the barley genome is a serious problem [13,133] and must be resolved.

Although resistances derived from *Hb* are based on major genes, it is believed [38] that some of them should be more durable than resistances originating from the primary gene pool. Cultivated as well as bulbous barley are infected with powdery mildew, but outside of Israel no cross infection was recorded [134], and the locations where *Hb* is found naturally and barley is cultivated do not overlap extensively, which might prevent a rapid adaptation of the pathogen to overcome resistances derived from *H*. *bulbosum*.

### 3.4. Utilization of the Tertiary Genepool

The tertiary genepool of cultivated barley comprises 29 species of *Hordeum* [127]. Twenty-six of them were inoculated with *Bgh* isolates, and all except an accession of *H*. *marinum* expressed imunity [135]. Two susceptible *H*. *vulgare* cultivars were inoculated with 287 isolates of *B*. *graminis* collected from *H*. *murinum* at 12 locations in southwestern Europe, and none were virulent to them [136]. Similarly, *H*. *chilense* was inoculated with four isolates of *Bgh* and expressed RT0 only [137]. It seems that *Blumeria* pathogens infecting species belonging to the tertiary genepool of *Hordeum* are distant from *Bgh*, and resistances derived from these *Hordeum* species and integrated into cultivated barley genome should be more durable. However, crossability barriers have so far precluded the use of species in the tertiary genepool being exploited.

### 3.5. Other Nonhost Resistances

Most organisms live in environments where many pathogens are present and are immune to most of them. Nonhost resistance to nonadapted pathogens is usually strong [138] and defined as immunity [139]. Therefore, there is an almost unlimited range of organisms that could be exploited. Additionally, some artificial molecular sequences causing resistances to pathogens could serve as nonhost resistances.

The genetics of nonhost resistance remain poorly understood but can be expected to be predominantly polygenic [140], and the response would be with nonhypersensitive reactions [141]. However, even nonhost resistance can be overcome as has occurred with resistance in triticale (*Triticosecale*) to *B. graminis*. This relatively new cereal crop is derived from an intergeneric hybrid between two cereal species—wheat and rye [142], both of which and triticale itself are grown in similar areas and attacked with powdery mildew. Therefore, it was no surprise that the resistance of triticale was overcome [143] with a pathogen that arose through the hybridization of *B*. *graminis* f. sp. *tritici* and *B*. *graminis* f. sp. *secalis* [144]. Hence, specific pathotypes of the pathogen [145,146] evolved and created a new host for powdery mildew.

This review is devoted mainly to specific resistances operating on the gene-for-gene basis [7,8]. However, recent research indicates that the genetic of *H. vulgare*-*Bgh* relations in infection–defense interactions is much more complex [147,148], operates in different phases of interactions and alters these processes [149,150]. Knowledge recently acquired about these aspects of plant pathology, therefore, makes this topic eminently suitable for a comprehensive review.

## 4. Conclusions

- Since a previous conspectus [21] several resistance genes of barley against powdery mildew have been described, and many more specific resistances have been detected in cultivars, landraces and wild barley.
- Knowledge of specific resistance genes in hosts including barley has wide utilization in further research and practice, e.g., when looking for partial resistance, for the study of evolution of pathogen populations or mapping and the distribution of native resistances worldwide. It is also a valuable tool to establish authenticity and genotype purity of cultivars and to confirm their pedigrees.
- The primary importance of resistance genes is the protection of a host against a pathogen. Effectiveness of specific resistances to avirulent pathotypes is often great, but is soon overcome by virulent pathotypes of the pathogen. As all existing reports relating to barley and powdery mildew confirm, specific resistances alone hardly contribute to sufficiently durable resistance of cultivars because there is no appropriate method of using them to obtain resistance durability.
- The nonspecific *mlo* gene can provide a source of durable resistance of spring barley cultivars.
- Pyramiding of nonspecific quantitative resistance genes or use of introgressions from bulbous barley are promising ways to achieve sufficient resistance durability in winter barley cultivars.
- A successful method of gaining durable resistance might be to exploit nonhost resistances, especially those originating from related species found in different areas, e.g., resistances of barley derived from rice [151], or from species naturally attacked by distantly related pathogens.
- Specific resistances can supplement and enhance genetic resistance using the above strategies of breeding barley, and their effectiveness is proportional to the frequencies of the corresponding avirulences in the pathogen population.
- Recommended strategies of breeding barley for genetic resistance against powdery mildew can be combined.

## Figures and Tables

**Table 1:** Previously described barley powdery mildew resistance genes [21].

Gene	Chromosome	Gene	Chromosome
*Mla1*	1H	*Mlat*	1H
*Mla2*	1H	*MlGa*	1H
*Mla3*	1H	*Mlk1^2^*	1H
*Mla5*	1H	*Mlk2*	1H
*Mla6*	1H	*Mlnn*	1H
*Mla7*	1H	*Mlra*	1H
*Mla8*	1H	*Mlg*	4H
*Mla9*	1H	*MlBo*	4H
*Mla10*	1H	*Mlh*	6H
*Mla11*	1H	*MlLa*	2H
*Mla12*	1H	*mlo* ^3^	4H
*Mla13*	1H	*Mla_ma_*	1H?
*Mla14*	1H	*Mlab*	
*Mla15^1^*	1H	*Ml(Ab)*	
*Mla16*	1H	*Mlci*	1H?
*Mla17*	1H	*Ml(CP)*	4H?
*Mla18*	1H	*mld*	
*Mla19*	1H	*Ml(He)*	
*Mla20*	1H	*Mli*	
*Mla21*	1H	*Ml(IM9)*	
*Mla22*	1H	*Mlkb*	
*Mla23*	1H	*Ml(LG4)*	
*Mla24*	1H	*Ml(Ma)*	
*Mla25*	1H	*Mlmu*	1H?
*Mla26*	1H	*Mlmw*	1H?
*Mla27*	1H	*Mln*	1H?
*Mla28*	1H	*Mlne*	
*Mla29*	1H	*mlni*	
*Mla30*	1H	*Mlp*	
*Mla31*	1H	*Mlr74*	
*Mla(Al2)*	1H	*Mlr81*	
*Mla(BR2)*	1H	*Ml(Ru2)*	
*Mla(Tu2)*	1H	*mls*	
*Mla(No3)*	1H	*mlw*	
*Mla(No4)*	1H	*Ml(Wo)*	
*Mla(LG2)*	1H	*Mlx*	
*Mla(LG3)*	1H	*Mly*	
*Mla(Mu2)*	1H	*Mlz*	
*Mla(Tr3)*	1H	*Ml501*	
*Mla(MC3)*	1H	*Ml(1192)*	
*Mla(MC4)*	1H	*Ml(2891)*	
*Mla(Du2)*	1H	*Ml(3576)*	
*Mla(Em2)*	1H		
*Mla(Ru3)*	1H		
*Mla(Ru4)*	1H		

^1^*Mla15* = *Mla7*; ^2^ Originally designated *Mla4*; ^3^ Twenty-five *mlo* genes (allels) are listed.

**Table 2:** Recently described resistance genes against barley powdery mildew.

*Ml*-Gene	Chromosome	Source^1^/Variety	Reference(s)
*aN81*	1HS	V, Nepal 81	[22]
*(Ba)*	Unknown	V, Banteng	[23]
*(Dr2)*	Unknown	V, Dura	[23]
*(Hu4)*	Unknown	V, Hulda	[23]
*(Kr)* ^2^	Unknown	V, Kredit	[9,23]
*(Pl2)*	Unknown	V, Paula	[23]
*(St)*	Unknown	V, Steffi	[23]
*(Ch)*	Unknown	V, CH-669	[24]
*(Dt6)*	Unknown	V, Duet	[24]
*(Ln)*	Unknown	V?, Landi	[25]
*aLo*	1HS	V, Lomerit	[26,27]
*Sb*	7HL	V, SBCC097	[28]
*(Lu)*	Unknown	V, Lunet	[19]
*Mor*	2HS	V, 2553-3	[29]
*La-H*	2HL	V, HOR2573	[30]
*Mlmr*	6HL	V, 173-1-2	[31]
*(VIR)*	Unknown	V, VIR6139	[27]
*a32*	1HS	S, 142-29	[32]
*F*	7HL	S, RS137-28	[33]
*J*	5HL	S, HSY-78	[33]
*mlt*	7HS	S, RS42-6	[33]
*(Ro)*	Unknown	S, 1B-53/Roxana	[34]
*aLv*	1HS	S?, Laverda	[27,35]
*(Ve)*	Unknown	S?, Venezia	[36]
*hb1*	2HS	B, 81,882/83	[37,38]
*hb2*	Unknown	B, C1-5	[39]
*hb.A42*	2HS	B, A42	[13]

^1^ V = *Hordeum vulgare* subsp. *vulgare*, S = *Hordeum vulgare* subsp. *spontaneum*, B = *Hordeum bulbosum*; ^2^ Originally designated 1192 [21].

## Supplementary Materials

The following are available online at https://www.mdpi.com/2073-4425/11/9/971/s1. Table S1: Response types developed on leaves of barley after inoculation with an isolate of *Blumeria graminis* f. sp. *hordei*.
