# Supplementary material for: Specific Resistance of Barley to Powdery Mildew, Its Use and Beyond: A Concise Critical Review
Source: Genes (Basel). 2020 Aug 21;11(9):971. doi: 10.3390/genes11090971 (PMC7565388; doi:10.3390/genes11090971)
Supplement: Supplementary file 1 [file genes-11-00971-s001.pdf]

Supplementary Table S1. Response types developed on leaves of barley after inoculation with an isolate of *Blumeria graminis* f. sp. *hordei*

Response type	Mycelium growth	Sporulation
0	None	None
0–1	None	None
1	Weak	None
1–2	Weak	Weak
2	Moderate	Weak
2–3	Moderate	Moderate
3	Strong	Moderate
3–4	Strong	Strong
4	Strong	Strong

[45] Modified.
